# Circular Subaperture Stitching Interferometry Based on Polarization Grating and Virtual–Real Combination Interferometer

**DOI:** 10.3390/s22239129

**Published:** 2022-11-24

**Authors:** Yao Hu, Zhen Wang, Qun Hao

**Affiliations:** 1 Beijing Key Laboratory for Precision Optoelectronic Measurement Instrument and Technology, School of Optics and Photonics, Beijing Institute of Technology, Beijing 100081, China; 2 School of Opto-Electronic Engineering, Changchun University of Science and Technology, Changchun 130022, China

**Keywords:** subaperture stitching, polarization grating, virtual–real combination interferometer

## Abstract

This paper presents a polarization grating based circular subaperture stitching interferometer. The system can be used for small F/# concave surface tests with a large F/# transmission sphere, where F/# is the ratio of focal length to aperture. A polarization grating was employed to deflect the incident beam for subaperture scanning by its axial rotation instead of a multi-axis motion-control system. Compared with the traditional subaperture stitching interferometric system, the system proposed in this paper is smaller in size and reduces the measurement error introduced by mechanical adjustment. Using a virtual interferometer model and a virtual–real combination algorithm to remove the retrace error, the full-aperture figure error can be directly obtained without the need for a complex stitching algorithm. The feasibility of the algorithm was verified, and the measurement error caused by the modeling error was analyzed by simulation. The capability of the polarization grating to scan subapertures was experimentally confirmed, and possible solutions to some engineering challenges were pointed out. The research in this paper has pioneering and guiding significance for the application of polarization grating in interferometry.

## 1. Introduction

Optical components such as lenses and mirrors are widely used in astronomy, space optics, military defense, high-tech civilian use, and other fields. Aspheric surfaces are more capable of correcting aberrations than spherical surfaces, allowing optical designs and systems to use fewer components, reducing system weight, volume, complexity, and increasing system transmittance. Due to the above advantages, aspheric components have become indispensable in various fields. Among them, the aspheric surface with small F/#, i.e., relatively larger aperture and shorter radius of curvature, has been mainly used in lithography objective lens [1], optical disc read head [2], illumination system [3], and conformal dome [4,5] because of the excellent performance. The high-precision measurement of its surface shape is one of the prerequisites for the application of aspheric surfaces. As a non-contact and highly accurate method, interferometry is critical for high-precision final inspection of aspheric surfaces.

Common aspheric full-field interferometry includes auto-collimation, computer-generated hologram (CGH), compensation lens, Sub-Nyquist interferometry, etc. The auto-collimation method [6,7,8] can achieve precise null tests for quadratic aspheric surfaces, and it will cause center occlusion for rotationally symmetric surfaces. However, small F/# aspheric surfaces are mostly rotationally symmetric in the application scenario, and the central area is more critical. This method will cause data to be missing, and thus the application scope is limited. CGH method [6,9,10] uses a CGH as the compensator to achieve high-precision null tests of various aspheric surfaces. However, a CGH is expensive and needs to be used in one-to-one correspondence with the surface under test (SUT), resulting in poor versatility. The CGH line width corresponding to the steep area of a small F/# aspheric surface is small, so the design and processing are difficult. The compensation lens method [6,11,12] can perform a null or non-null test on aspheric surfaces. The null test with a null lens has similar deficiencies to the CGH method. In contrast, partial compensators in the non-null test do not need to compensate for all aspherical aberrations, so the design and processing difficulty of the compensator is slightly reduced, and its versatility is improved. Still, the interferometer requires a higher lateral resolution to resolve the high-density fringes generated by residual wave aberrations. In the case where aspheric surfaces with a small F/# usually have high-order aberrations and large slope asphericity [13,14], the contradiction between the above two is exacerbated, so the compensation lens method may need to face great difficulty in design and processing and high-density fringe resolution at the same time. Sub-Nyquist interferometry [6,15] can handle extremely dense fringes and use a transmission sphere (TS) to test aspheric surfaces with higher slope asphericity. It eliminates the requirement of the lateral resolution of the interferometer to a certain extent, but it needs to be matched with a special detector and an unwrapping algorithm. The former is easily affected by high-frequency noise, and the latter requires prior knowledge about the measured surface, so the application is limited.

In addition, F/# and R/D are usually positively correlated, where R is the radius of curvature of the vertex of the SUT and D is the clear aperture. As a common requirement for the full-field testing of spherical or aspheric SUTs in a single test, the F/# of the TS or compensator needs to be smaller than the R/D of the SUT. However, a TS or compensator’s design difficulty and manufacturing cost significantly increase with its F/# decrease.

Therefore, accurate interferometric testing for large-diameter and small F/# aspheric surfaces has always been a challenge for the full-field interferometric testing approaches due to the design and processing difficulty of the compensator, the limitation of the resolution of the interferometer, and the F/# of the TS.

Subaperture stitching interferometry (SSI) is a solution proposed in the 1980s [16,17,18,19,20,21,22,23,24,25,26]. SSI divides the SUT into several subapertures whose R/D is within the large F/# TS test range and measures them in sequence, thereby avoiding the challenges mentioned above. Then, the figure errors of different subapertures are stitched to obtain the full-aperture figure error of the SUT. According to the different subaperture shapes, SSI can be divided into annular subaperture stitching interferometry (ASSI) [22,23,24] and circular subaperture stitching interferometry (CSSI) [16,17,18,19,20,25,26]. ASSI is designed for rotationally symmetric aspheric surfaces, and its subapertures are usually multiple concentric rings. Using a TS to generate spherical waves with different curvature radii to match the slopes of different annuli on the SUT, ASSI mainly expands the longitudinal measurement range of the interferometer. Zygo Corporation has commercial instruments that enable ASSI measurements. CSSI uses spherical waves of different curvatures to match the local surface shapes of the measured surface within the range of different circular subapertures. It greatly expands the lateral and longitudinal measurement ranges of the interferometer and can achieve high-resolution tests of large-aperture plane, spherical, aspheric, and freeform surfaces without using a null lens. It is now commercially available by QED Technologies Corporation.

However, SSI still faces new challenges in terms of the device structure, stitching algorithm, and error correction.

First of all, the ASSI measurement process requires a high-precision axial control and measurement device, the range of which is no less than the sag of the SUT, while CSSI usually requires the use of a bulky and complex multi-axis motion control system to realize the relative movement between the SUT and the interferometer. In addition, multiple adjustment degrees of freedom inevitably introduce mechanical errors during the calibration process, which become an important factor limiting the test accuracy. Developing and introducing more compact scanning mechanisms with fewer sources of errors are required.

Secondly, no matter the kind of SSI, the stitching algorithm that fuses different subapertures to obtain full-aperture figure error plays an important role. Many landmark algorithms have been proposed for subaperture stitching, such as Kwon Thunen method [17], simultaneous fit method [27], discrete phase method [21,28], multi-aperture overlap-scanning technique [29], and the subaperture stitching and localization algorithm [30]. However, they all rely on complex mathematical calculations for positioning and inconsistency calibration between subapertures, which are prone to introduce positioning and calculation errors. Algorithms for data stitching without calibration need to be explored.

Finally, most SSIs are non-null interferometry, which means a retrace error [31,32] caused by the measurement light not returning in the same way the incident light will always exist. However, most of the above algorithms do not treat the retrace error suitably. Therefore, in order to ensure sufficiently high measurement accuracy in the absence of retrace error removal, the subaperture needs to be sufficiently small, which leads to an increase in the number of tests, larger stitching cumulative error, and a greater negative effect from the environment, or that the subapertures are equipped with a variable compensator, which increases the complexity of the system. Therefore, it is necessary to deal with the retrace error to obtain a high-precision aspheric figure error result. Gappinger et al. [33] modeled a non-null interferometric system and used the reverse iterative optimizing reconstruction (ROR) algorithm to calibrate the non-null interferometric results for the first time. Zhang et al. [34,35] proposed a simultaneous reverse iterative optimizing reconstruction (SROR) algorithm for subaperture stitching, modeled the optical wedge scan interferometric system [36], and analyzed the influence of interferogram noise and modeling error on test accuracy. To address the influence of retrace error and alignment error in practical interferometers, a virtual–real combination interferometer was proposed in our previous work [37,38] as a tool to describe optical systems and eliminate system errors accurately.

Aiming at the above problems of SSI, this paper proposes a polarization grating based circular subaperture stitching interferometer (PG-CSSI) with a virtual–real combination algorithm for small F/# concave surface tests with a large F/#TS. In the interferometric system, we propose that a polarization grating (PG) is employed to deflect the incident beam [39,40,41] for subaperture scanning by the PG’s axial rotation instead of a multi-axis motion-control system, which greatly reduces the system’s volume, the degree of freedom, and error sources. Aiming at the problems of stitching error accumulation and retrace error correction in SSI, this method combines a virtual–real combination interferometer with the SROR algorithm to reconstruct the figure error. This method concurrently eliminates the retrace errors introduced by the non-null test and additional components, requiring neither a high overlap ratio of the subapertures nor complex stitching algorithms, thus reducing positioning and calculation errors. The remainder of the paper is arranged as follows. In Section 2, the principle of the PG-CSSI system and the virtual–real combination interferometer are outlined; in Section 3, the feasibility of the system is verified, and the influence of modeling errors is analyzed by simulation; Section 4 verifies the capability of PG to scan subapertures and discusses possible solutions to some engineering challenges; Section 5 gives the conclusion.

## 2. Methods and Theory

### 2.1. Principle and Design of PG-CSSI

**Figure 1:**
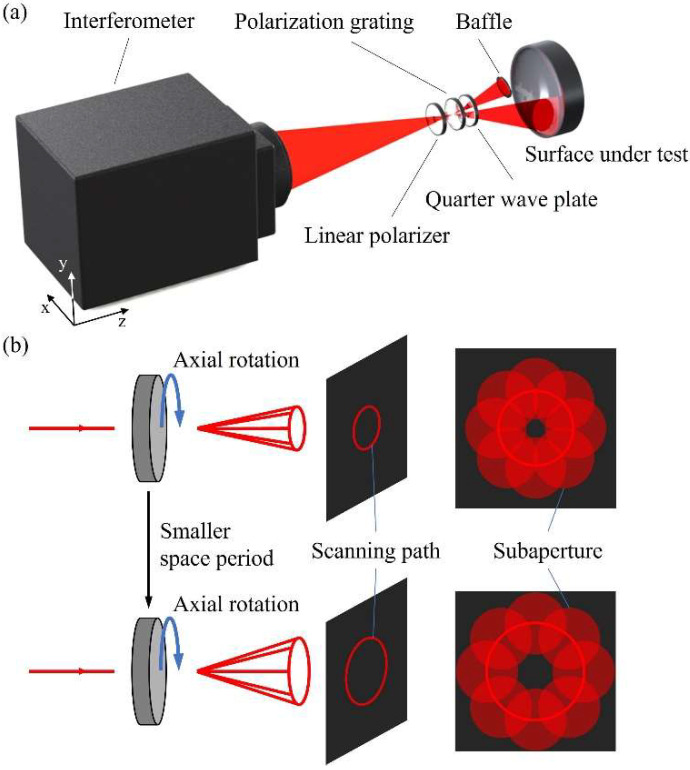
(**a**) Schematic diagram of PG-CSSI. (**b**) The function of PG in PG-CSSI.

Figure 1a shows the schematic diagram of PG-CSSI. The large F/# spherical wave, emitted by an interferometer, travels through a linear polarizer (LP). It converges on the front surface of the PG, which diffracts linearly polarized light into ±1st order light. One beam is blocked by a baffle, and the other travels through a λ/4 wave plate (QWP) and is reflected by the SUT, forming the test beam after traveling back to the interferometer. The axial position of the SUT can be determined according to its closest comparison sphere [42,43], and the exact method will not be repeated here. PG-CSSI realizes the off-axis subaperture scanning by the axial rotation of the PG, while the central subaperture is an ordinary spherical SUT test system, requiring no LP, PG, or QWP.

The key element of PG-CSSI is PG, whose function is to make the required deflection of the incident beam and to ensure that the wavefront distortion is as small as possible, and that the diffraction efficiency is as high as possible. Compared with ordinary diffraction gratings, which usually have 0th order and high orders diffracted light, the main advantage of PG is that it can theoretically reduce the diffraction efficiency of other orders to 0, except for the ±1st orders. Therefore, PG can ensure that no other unwanted orders exist in interferometry and that the diffraction efficiency of the order used for the measurement is high. As shown in Figure 1b, the PG deflects the incident beam to measure the off-axis subaperture regions, and its axial rotation can achieve subaperture scanning in the annular region. As an extension, more peripheral areas can be tested using a PG with a smaller spatial period, enabling multi-ring scanning. Compared with the multi-axis motion-control system, PG can realize the subaperture scanning test through only one degree of freedom, namely its axial rotation, greatly reducing both the system volume and the measurement error introduced by the adjustment degree of freedom.

**Figure 2:**
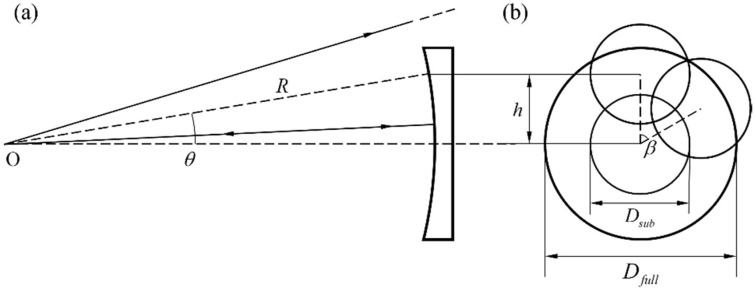
(**a**) Optical path diagram of the off-axis subaperture (between the focus O of the TS and the SUT). (**b**) Schematic diagram of the subaperture layout. O is the focus of the TS. *θ* is PG’s deflection angle to the chief ray. *R* is the radius of curvature of the SUT. *h* is the center distance between the off-axis subaperture and the center subaperture. *β* is the rotation stepping angle of the PG. *D*_sub_ is the diameter of the central subaperture. *D*_full_ is the diameter of the SUT.

As shown in Figure 2, the core parameters of PG include its deflection angle *θ* to the light, the axial rotation stepping angle *β* during the scanning process, and the diffraction efficiency *η*. The first two are usually determined by the functional parameters of the system, such as the F/# of the TS, wavelength *λ*, and surface parameters of the SUT. Taking a spherical surface as an example, as shown in Figure 2a, the center distance *h* between the off-axis subaperture and the center subaperture satisfies

(1) h=Rsinθ,

where *R* is the radius of curvature of the SUT. The diameter of the central subaperture is easily determined by

(2) $$D_{\mathrm{sub}}=R/F,$$

where *F* is the F/# of TS. As shown in Figure 2b, the included angle between the lines connecting the center of two adjacent off-axis subapertures to the center of the SUT is the rotation stepping angle *β* of the PG. In traditional SSI, the overlap ratio of subapertures is determined by *D*_sub_, *h*, and *β*, and is usually required to reach more than 25%. Thanks to the virtual–real combination algorithm proposed in Section 2.2, PG-CSSI has no such requirement and only needs to ensure that all subaperture can cover the full aperture, which results in a larger *h* or *β* and thus a higher scanning efficiency given the same *D*_sub_. According to the full coverage requirement, *h* and *β* can be determined in combination with the help of *D*_sub_, and then *θ* can be obtained by Equation (1). The deflection angle *θ* of the light is the diffraction angle of the normal incident light on the PG, which follows the grating equation

(3) Λsinθ=λ,

where *Λ* is the space period of PG. If the wavelength *λ* of the interferometer is not easy to adjust, the deflection angle *θ* can be adjusted to the desired value by changing *Λ*.

**Figure 3:**
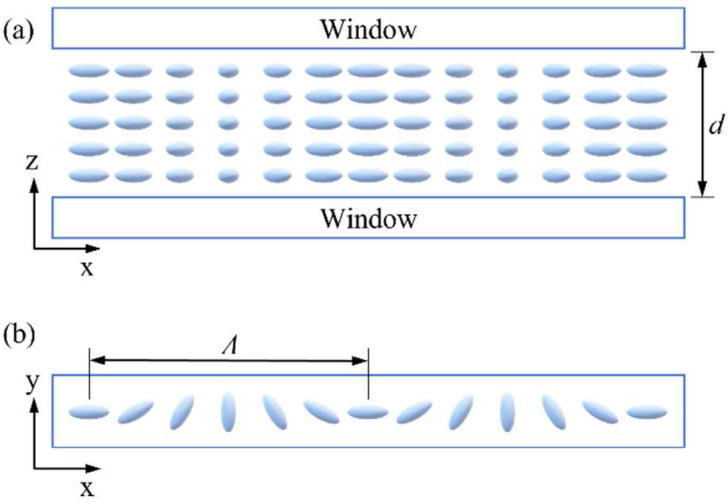
(**a**) Front-view and (**b**) top-view of the microstructure of PG. The blue rod-like structures in the figure are LC molecules. *d* is the thickness of the LC layer. *Λ* is the space period of PG.

The diffraction efficiency *η* of PG can be explained using the geometric phase principle [44,45,46]. When a phase delay element is illuminated by a light formulated as $E_{\mathrm{in}}={\left[1\mp j\right]}^{T}$, the Jones vector of the output light can be expressed as

(4) $$E_{\mathrm{out}}=\cos\left(\frac{\delta }{2}\right)\left[\begin{array}{c} 1\\ \pm j \end{array}\right]-j\sin\left(\frac{\delta }{2}\right)\exp\left(\pm j2\varphi \right)\left[\begin{array}{c} 1\\ \mp j \end{array}\right],$$

where *φ* is the local direction of the slow axis and *δ* is the phase retardation of the wave plate. The PG used in this paper is composed of a liquid crystal (LC) layer poured between two windows. The microstructure is shown in Figure 3. The slow axis rotates along the x-axis with an angle, and *d* is the thickness of the LC layer.

To investigate the diffraction behavior of such a grating in the paraxial domain, the far-field electric field of the *m* diffraction order after the Fourier transformation can be calculated as

(5) $$D_{m}=\frac{1}{\Lambda }\int_{0}^{\Lambda }T\left(x\right)E_{in}\exp\left(-j2\pi mx/\Lambda \right)dx,$$

where the local Jones matrix can be given by

(6) $$T\left(x\right)=R\left(-\frac{\pi x}{\Lambda }\right)\left[\begin{array}{cc} \exp\left(-j\delta /2\right) & 0\\ 0 & \exp\left(j\delta /2\right) \end{array}\right]R\left(\frac{\pi x}{\Lambda }\right),$$

where **R** is the rotation matrix. The diffraction efficiency of the *m* order is

(7) $$\eta_{m}=\frac{{\left|D_{m}\right|}^{2}}{{\left|E_{in}\right|}^{2}}.$$

Simplify Equation (5) as

(8) $$D_{m}=\Gamma_{m}E_{in},$$

where the grating transfer matrix

(9) $$\Gamma_{m}=\frac{1}{\Lambda }\int_{0}^{\Lambda }T\left(x\right)\exp\left(-j2\pi mx/\Lambda \right)dx$$

has non-zero solutions only for 0 and ±1 orders. The corresponding diffraction efficiency can be calculated from Equation (7) as

(10) $$\eta_{0}=\cos^{2}\left(\delta /2\right),$$

(11) $$\eta_{\pm 1}=\frac{1}{2}\left(1\mp {S^{\prime }}_{3}\right)\sin^{2}\left(\delta /2\right)$$

where *δ* = 2*π*Δ*nd*/*λ* is the phase retardation of the LC layer, Δ*n* is the birefringence of LC, *λ* is the wavelength of the incident light, and ${S^{\prime }}_{3}=S_{3}/S_{0}$ is the normalized Stokes parameter related to the ellipticity of input light.

It can be seen that only 0 and ±1 orders exist. The +1 and −1 orders are left- and right-handed circular polarization, respectively. The 0th-order diffraction efficiency can be tuned by controlling the phase retardation, which relies on the type of LC material and its layer thickness. The theoretical *η*_0_ of PG used in this paper is zero, so only ±1st orders diffracted light exist. Ordinary diffraction gratings cannot achieve such performance. Because PG can ensure that no other unwanted orders exist in interferometry and can achieve any scanning angle *θ* by changing the period *Λ*, it has a huge advantage in subaperture scanning compared to ordinary diffraction gratings.

So far, the system structure of the PG-CSSI proposed in this paper has been introduced, as well as the working principle and parameter selection of its key component, PG. With the above system, we can realize wavefront measurements for the 1 + N subapertures, including the central and the off-axis subapertures. The system does not require a complex multi-axis motion control system and only requires the axial rotation of the PG around the axis to scan and measure the off-axis area, which not only greatly reduces the system volume but also reduces the adjustment degrees of freedom and error sources. The following section introduces the principle of completing the full-aperture figure error measurement through the virtual–real combination interferometer.

### 2.2. Virtual–Real Combination Algorithm in PG-CSSI

The system mentioned above has no compensator, so there naturally are retrace errors when measuring aspheric surfaces. Introducing flat glass components and diffractive components such as LP, PG, and QWP in the non-collimated optical path will increase wavefront aberration, increasing the retrace error of off-axis subaperture tests. Hence, the image plane wavefront obtained by processing the interferogram cannot be regarded as twice the figure error of the SUT. Therefore, a virtual–real combination interferometer [37,38] is introduced to obtain the figure error according to the wavefront measurements.

**Figure 4:**
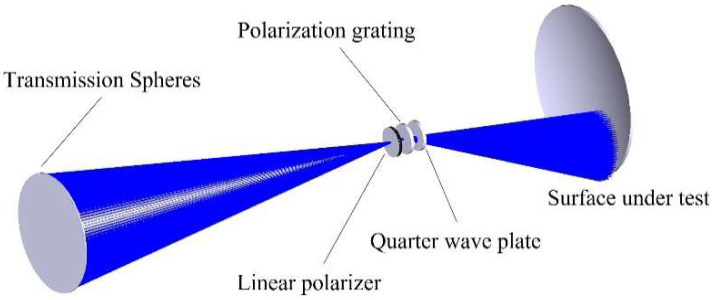
Schematic diagram of the virtual interferometer.

First, a virtual interferometer needs to be modeled. The optical path parameters of the test arm in the real interferometer, such as component thickness and distance, were accurately measured and then used to model the virtual interferometer shown in Figure 4 by ray tracing. The structure of the virtual interferometer is the same as that of the real interferometer, except that the SUT is an ideal surface without figure error. In order to simplify the model, the internal structure and error of the interferometer are ignored, and an ideal paraxial lens of the corresponding aperture and focal length is used to simulate the TS. It is worth noticing that if the virtual and the real interferometer are not exactly inconsistent, the introduced modeling error will cause further measurement errors. To address that, a simulation with analyses is performed in Section 3.2.

After modeling the virtual interferometer, optimization needs to be performed in it to obtain the figure error. Since multiple subapertures are required to cover the entire aperture of the SUT, if each subaperture is optimized in sequence and then stitched together, it will not only be time-consuming but will also introduce additional positioning errors and stitching algorithm errors. Therefore, we take full advantage of the virtual–real combination interferometer and use the SROR algorithm [36]. A virtual interferometer with multiple configurations is set up in the ray tracing program, and each configuration characterizes a subaperture measurement. Only the axial rotation angle of the PG is different in all the interferometric configurations, corresponding to different subaperture areas on the SUT. In addition, the central subaperture is an ordinary spherical SUT test setup, requiring no LP, PG, or QWP. The image plane wavefronts of the real and virtual interferometric systems can be expressed as implicit non-linear functions

(12) $$\{\begin{array}{c} W_{n}=f_{n}\left(E_{\mathrm{fig}-n}+E_{\mathrm{rt}-n}\right)\\ {W^{\prime }}_{n}=g_{n}\left({E^{\prime }}_{\mathrm{fig}-n}+{E^{\prime }}_{\mathrm{rt}-n}\right) \end{array}\;\;,$$

where $E_{\mathrm{fig}-n}$ and $E_{\mathrm{rt}-n}$ are the figure error and retrace error in the real interferometer, while ${E^{\prime }}_{\mathrm{fig}-n}$ and ${E^{\prime }}_{\mathrm{rt}-n}$ are the counterparts in the virtual one, respectively. *n* represents the *n*th subaperture. A merit function is defined as

(13) $$\begin{array}{cc} MF & ={\left(E_{\mathrm{fig}-n}-{E^{\prime }}_{\mathrm{fig}-n}\right)}^{2}\\ & ={\left[\left(f_{n}^{-1}\left(W_{n}\right)-E_{\mathrm{rt}-n}\right)-\left(g_{n}^{-1}\left({W^{\prime }}_{n}\right)-{E^{\prime }}_{\mathrm{rt}-n}\right)\right]}^{2}, \end{array}$$

when the real and virtual interferometers are consistent, we have

(14) $$E_{\mathrm{rt}-n}={E^{\prime }}_{\mathrm{rt}-n}.$$

Therefore, the merit function in Equation (13) can be simplified as

(15) $$MF={\left[f_{n}^{-1}\left(W_{n}\right)-g_{n}^{-1}\left({W^{\prime }}_{n}\right)\right]}^{2}.$$

In order to facilitate the optimization process, Equation (15) can be considered as

(16) $$MF={\left(W_{n}-{W^{\prime }}_{n}\right)}^{2}.$$

Equations (15) and (16) are physically equivalent. That is, Equation (15) is considered to reach its minimum value when Equation (16) reaches its minimum value. For a clearer definition, we employed the Zernike polynomial to describe the image plane wavefront and figure error. Equation (16) can be written as

(17) $$MF=\sum_{i}{\left(Z_{i}-{Z^{\prime }}_{i}\right)}^{2},$$

where Z and $Z^{\prime }$ are the Zernike coefficients employed to describe the subaperture image plane wavefronts of the real and virtual interferometer, respectively. *i* is the index of the Zernike polynomial term.

If all the subaperture image plane wavefronts in the virtual interferometer are close enough to those in the real one, the full aperture figure error in the virtual interferometer would be able to characterize the actual one. Therefore, the optimization is implemented on all the subapertures simultaneously in the multiple configurations rather than each subaperture separately. A global merit function is defined according to Equation (17) as

(18) $$MF=\sum_{n}\sum_{i}{\left(Z_{ni}-{Z^{\prime }}_{ni}\right)}^{2}.$$

The Zernike polynomials for different subapertures are normalized to the subaperture and not to the full aperture. Taking the full aperture figure error in the virtual interferometer ${E^{\prime }}_{\mathrm{FIG}}$ as the optimization variable, the optimization process is as follows. The initial value of ${E^{\prime }}_{\mathrm{FIG}}$ is zero. The optimizer continuously changes the value of ${E^{\prime }}_{\mathrm{FIG}}$ and calculates its corresponding image plane wavefront ${W^{\prime }}_{n}$ in the ray tracing program to make ${W^{\prime }}_{n}$ approach the actual one $W_{n}$. When Equation (18) reaches the minimum value, ${W^{\prime }}_{m}$ can be regarded as equal to $W_{m}$, so that ${E^{\prime }}_{\mathrm{FIG}}$ can be regarded as equal to the actual $E_{\mathrm{FIG}}$. The damped least squares method can be used for optimization.

## 3. Simulation and Discussions

In this section, simulations are carried out to verify the feasibility and stability of PG-CSSI based on virtual–real combination interferometer. In Section 3.1, SUTs with different surface parameters and the same figure error were tested to verify the feasibility of the virtual–real combination SROR algorithm proposed in this paper. In Section 3.2, the influence of the modeling error on the test results of the figure error was analyzed to guide the experimental process.

**Figure 5:**
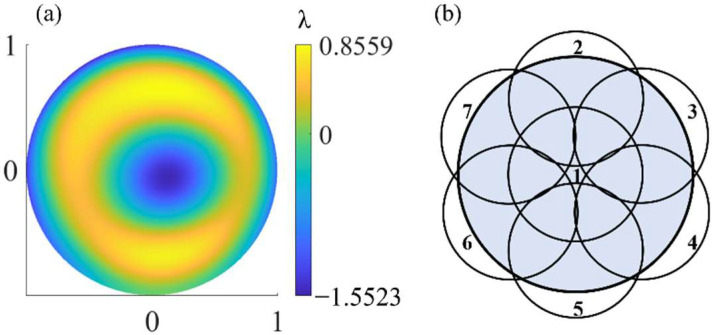
(**a**) True figure error. (**b**) Schematic diagram of subaperture layout.

Two Fizeau interferometers were simulated—one as the real interferometer to generate the measured image plane wavefront and the other as the virtual interferometer to complete the measurement with the algorithm in Section 2.2. As mentioned, each of the Fizeau interferometers were modeled in the ray tracing program using multi-configuration, in which each configuration characterizes a subaperture measurement. Only the axial rotation angle of the PG is different in all the interferometric configurations, that is, in the process of off-axis subaperture scanning, the PG-CSSI has only this one adjustment degree of freedom. We used a paraxial lens to simulate the TS of the interferometer. The full-aperture figure error added to the SUT in the simulated real interferometer is shown in Figure 5a. The figure error is a distribution obtained by fitting the figure error test results of an actual spherical mirror using 36-term Zernike standard polynomials, with a PV value of 2.41λ and an RMS value of 0.58λ. It has an overall characteristic of rotational symmetry with some local fluctuations, which conforms to the figure error distribution that may exist in the machining process of the rotationally symmetric surface. The number of terms of the used Zernike polynomial meets the requirements of the spatial frequency in practical interferometry. The spatial resolution of the simulated image plane wavefront in the real interferometer is 512 × 512.

In order to reduce the actual measurement and optimization time and improve the measurement efficiency, as few subapertures as possible were used to realize the measurement. The simulation uses one central subaperture and six off-axis subapertures to ensure full coverage of the full aperture of the SUT, as shown in Figure 5b, that is, the axial rotation stepping angle of PG *β* = 60° during the off-axis subapertures scanning process. The interferometer lens F/# and the spatial period *Λ* of the PG were adjusted accordingly to match the surface parameters of the SUT.

### 3.1. Feasibility with Different Surface Parameters

In order to verify the feasibility of the method proposed in this paper for spherical and rotationally symmetric aspheric surfaces test, SUTs with different F/# and K were tested, where K is the conic constant. Since the F/# of the rotationally symmetric aspheric surface is proportional to its R/D, for the convenience of calculation, R/D is taken as the characterization parameter. The details are shown in Table 1. Four spherical surfaces with different R/D and eight aspheric surfaces with different K values were measured, among which the aspheric surfaces included all quadric types. The minimum R/D of the measured sphere reaches 1, which means that the F/# of the TS needs to be smaller than 1 to achieve a common full-field measurement. The slope asphericity of the aspheric SUT reaches the maximum value of 0.0022 in the case of R/D = 2.5 and K = 2, which means that even if it can be full-field covered with a 4-inch TS with F/# less than 2.5, the minimum phase resolution requirement of 4 pixels/stripe cannot be met at CCD resolutions as high as 1200 × 1200. On this basis, it is also necessary to exclude the influence of large retrace errors. All in all, some of the SUTs in Table 1 can hardly be tested with full-field interferometry.

In order to use the subaperture layout shown in Figure 5b to achieve full coverage measurement, TS with different F/# and PG with different spatial periods are used to match the R/D of the SUT. Due to the different optical path structures, the subaperture overlap ratios in the cases listed in Table 1 are also different, ranging from 14% to 17%, which are much smaller than the overlap ratio required by traditional SSI.

**Figure 6:**
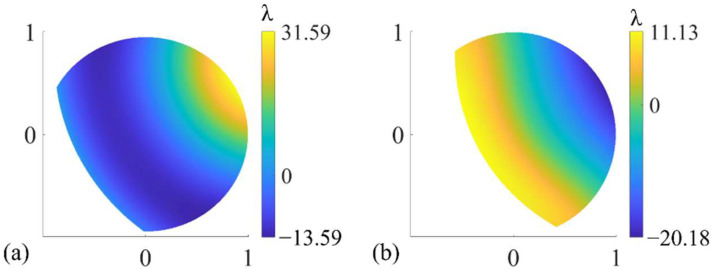
The simulated image plane wavefront of subaperture No. 6 of the (**a**) aspheric surface with R/D = 2.5, K = 2, and (**b**) spherical surface with R/D = 1.

Firstly, based on the simulated real interferometer, the wavefront measurement of each subaperture is performed, in which significant retrace error can be observed. The typical results are as follows. It can be seen from Figure 6a that the image plane wavefront of subaperture No. 6 of the aspheric SUT with R/D = 2.5 and K = 2 has a PV value of 45.18λ, which is much larger than the full-aperture figure error PV value 2.41λ in Figure 5a. The distribution is also completely different from the figure error of the corresponding subaperture, which intuitively shows that when the SUT is aspheric, there is naturally a retrace error in the system due to the fact that the light does not return in the original way. In addition, even if the SUT is spherical, a retrace error is introduced by components such as PG in the PG-CSSI. As shown in Figure 6b, the image plane wavefront of subaperture No. 6 of a spherical surface with R/D = 1 has a PV value of 31.31λ, which is also much larger than the PV value of the full-aperture figure error.

**Figure 7:**
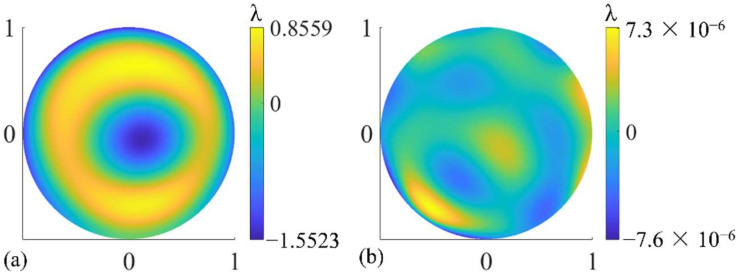
(**a**) The test result of the figure error of the SUTs with different surface parameters. (**b**) The difference between the test results and the true value.

As mentioned in Section 2.2, the simulated measured wavefront of each subaperture was characterized with Zernike polynomials and adopted to construct the merit function in Equation (18). The figure error of the SUT is described by 36-term Zernike polynomials as the optimization variable. After optimization, the value of the merit function can be reduced to 10^−8^ or lower. The specific test results are shown in Figure 7 and Table 1. The distribution of the test results in each case and the difference between them and the true value are basically the same, so only the result in the case where F/# is the smallest, corresponding to R/D = 1, K = 0, is given in Figure 7. It can be seen from Table 1 that the PV and RMS values of the test results can be regarded as consistent with the true value within the allowable error range. The PV and RMS values of test error are both in the order of 10^−5^λ and below.

Among the SUTs, the first four cases are spherical surfaces with different R/D. For conventional interferometry, the difficulty and error of the measurement usually decreases with the F/# or R/D, but the accuracy of our method has no significant change, which effectively shows the applicability of the method to different F/#. The last eight cases are aspheric, and it can be seen from the test results that the SROR algorithm can eliminate the retrace errors without affecting the measurement accuracy. As mentioned above, the subaperture overlap ratios of different SUTs are different, as shown in Table 1. This parameter usually affects the calculation accuracy in the traditional subaperture stitching algorithm. The PG-CSSI based on virtual–real combination interferometer can achieve high-precision tests under different subaperture overlap ratios that are lower than those required by traditional SSI. Therefore, the simulation confirms the feasibility of the system and method proposed in this paper and its advantages over the traditional SSI.

### 3.2. Modeling Error Tolerance Analysis

The above results are obtained under ideal conditions without any errors in the modeling of the virtual interferometer. In the real experiment, PG-CSSI may face various error sources, some of which are common in interferometry, such as wavelength fluctuation of the light source [47,48], inhomogeneity of the transmission element refractive index [49], air disturbance, reference surface figure error, detector noise, phase shift error, etc. The other part is unique to this method, such as the modeling error. The virtual–real combination interferometer is implemented based on system modeling, so its test accuracy will be affected by modeling errors. In the real experiment, the modeling error mainly comes from the alignment error and manufacturing errors of the components in the system. In order to guide the experimental process, this section analyzes the influence of component alignment and manufacturing errors on the test result. In order to explore the general law, taking a typical spherical measurement as an example, we first analyzed the typical alignment and manufacturing errors that may exist in the system, obtaining the variation of the image plane wavefront and the main affected aberrations caused by these errors. The errors introduced by the above aberrations to the test results and the tolerance were then analyzed.

A TS with 7.1/# and a PG with an 8 µm space period were selected to test the spherical SUT with the parameters of R/D = 4, K = 0. Inferred from the component manufacturing tolerance and assembly way of the components and the alignment process in the experiment, the alignment error and manufacturing errors of the components in the system and their maximum possible values are shown in Table 2. Among them, the axial and lateral translation errors are the estimated values of the alignment error with naked eyes. In the real experiment, LP, PG, and QWP were integrated into a sleeve through the retaining ring, so the tilt errors come from the parallelism error of the retaining ring. During the alignment of the SUT, the influence of the lateral translation and tilt of the SUT on the tilt term in the interferogram will compensate for each other. Thus, in Table 2, the tilt error of the SUT is set to a value that can compensate for its lateral translation error. The axial rotation error of the PG is the sum of the rotation error of the rotation mount and the estimated value of the adjustment error with naked eyes. The parallelism error of each element is the parallelism error of the protection windows in the sandwich structure.

**Figure 8:**
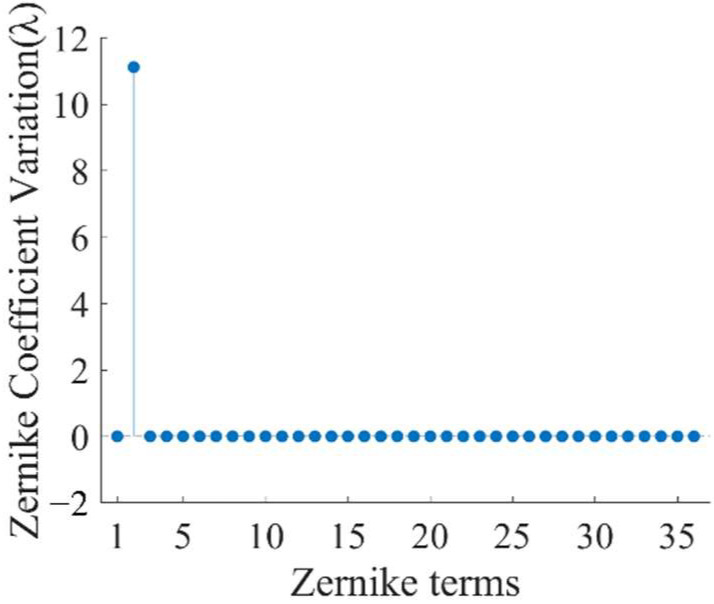
Variations of the Zernike coefficients describing the image plane wavefront of the subaperture No. 3 in Figure 7b caused by the SUT’s lateral translation error.

Each alignment error and manufacturing error were individually added to the real interferometer in the simulation, and the virtual interferometer was still in an ideal state. This operation will introduce modeling errors into the virtual interferometer. The variation $\Delta z_{i}$ between the Zernike coefficients describing the image plane wavefront of the real interferometer and the ideal virtual one can be obtained as the influence of the modeling error on the image plane wavefront, where *i* still represents the index of the Zernike polynomial term. For example, Figure 8 shows the variation of the Zernike coefficients describing the image plane wavefront of subaperture No. 3 in Figure 5b caused by the SUT’s lateral translation error of 0.5 mm. The variation is mainly tilt aberration, which coincides with physical intuition.

By adding the typical errors in Table 2 to the simulated real interferometer with an ideal SUT individually, we can obtain the variation of the Zernike coefficients describing the off-axis subaperture image plane wavefront caused by each alignment and manufacturing error, as shown in Table 3. Since The variation of the same Zernike coefficient of the image plane wavefront caused by the same alignment error in different subapertures is different in numeric values, but the same in the order of magnitudes. For the sake of simplicity, only the order of magnitudes are listed in Table 3. In addition, the ratios of the variation of the 9–36th terms of the Zernike coefficients to the variation of other terms are less than 1%, so they are not listed.

As can be concluded from Table 3,

1. Each alignment or manufacturing error has an obvious impact only on the low-order aberration of the image plane wavefront;
2. The greatest influence on the tilt aberration comes from the lateral translation or tilt error of the SUT;
3. The greatest influence on the power aberration comes from the axial translation error of the LP or the SUT;
4. Only the axial rotation error of the PG has an obvious impact on astigmatism and coma aberrations, and the order of magnitude is small.

So far, we have analyzed the influence of alignment and manufacturing errors on the Zernike coefficient describing the image plane wavefront. In order to further analyze the errors introduced by the image plane wavefront change mentioned above to the test result of the figure error, that is, the errors introduced by the optimization object *Z_ni_* (*i* = 2~8) error in Equation (18) to the test results, we changed *Z_ni_* (*i* = 2~8) to *Z_ni_* + Δ*Z_i_* in turn. The results are as follows.

Table 4 shows the figure error test results under different Δ*Z_i_* (*i* = 2~4) and their point-to-point differences with the true value. Among them, Δ*Z*_2_ = 10λ, Δ*Z*_3_ = 10λ, Δ*Z*_4_ = 1λ is the maximum possible variation that Δ*Z_i_* (*i* = 2~4) can take in the real experiment according to Table 3, respectively. In these cases, there is a large error in the test results of surface figure error. When Δ*Z*_2_, Δ*Z*_3_, Δ*Z*_4_ are reduced to 1λ, 1λ, and 10^−2^λ, respectively, the PV and RMS of the test error are, respectively, 1% of the total PV and RMS of the figure error and below. It is worth noticing that power aberrations have a greater impact on test results than tilt aberrations.

**Figure 9:**
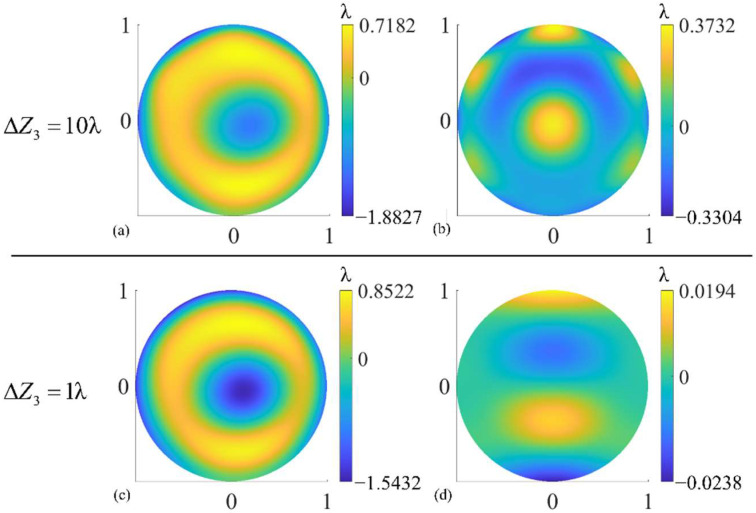
(**a**,**c**) Figure error test results under different Δ*Z*_3_ and (**b**,**d**) the test errors. Δ*Z*_3_ is the error of optimization object *Z*_3_.

It also can be seen from Table 4 that, compared with Δ*Z*_2_, the same order of magnitudes of Δ*Z*_3_, which is also tilt aberration, introduces a larger error to the test results. Besides, in the case of Δ*Z*_2_ and Δ*Z*_4_, the spatial distribution of test errors is irregular but similar for different values of Δ*Z_i_*, but a larger Δ*Z*_3_ introduces an obvious high-order aberration to the test results compared with a smaller Δ*Z*_3_, as shown in Figure 9. These phenomena may be because two subapertures, subaperture No. 2 and No. 5 in Figure 5b, are located in the *y* direction, but no subapertures are located in the *x* direction. These two subapertures are more sensitive to Δ*Z*_3_, which causes *y* tilt, than Δ*Z*_2_, which causes *x* tilt, and have a greater impact on the optimization results when performing SROR.

It can be seen from the above results that when Δ*Z_i_* (*i* = 2~4) of the image plane wavefront takes the maximum value in Table 3, great errors will exist in the test results. Further, Table 3 shows that Δ*Z*_2_ and Δ*Z*_3_ are mainly introduced by the alignment error of the SUT, and Δ*Z*_4_ is mainly introduced by LP-Δ*z* and SUT-Δ*z*. On the contrary, if the three aberrations Δ*Z_i_* (*i* = 2~4) are at the magnitude of the errors introduced by the other components, except for the SUT and LP, test results that are highly consistent with the true value can be obtained.

Therefore, if the alignment and manufacturing errors are not considered in the virtual interferometer modeling, it is necessary to precisely align the LP and the SUT in the real interferometer to ensure the accuracy of the measurement. If only SUT-Δ*x* and SUT-Δ*y* are considered, in order to control Δ*Z*_2_ and Δ*Z*_3_ within 1λ, SUT-Δ*x* and SUT-Δ*y* need to be reduced to at least 0.01 mm. If only LP-Δ*z* and SUT-Δ*z* are considered, in order to keep Δ*Z*_4_ within 0.01λ, the two errors must be reduced to at least 5 µm. The actual situation is usually more complicated, considering that the aberrations introduced by the components will couple with each other. It is difficult to realize such a high control accuracy in actual implementation, so in order to reduce the control difficulty and not reduce the accuracy of the measurement, an error correction procedure is suggested to be introduced in the data processing process.

The analysis continues below for different Δ*Z_i_* (*i* = 5~8). Table 5 shows the figure error test results under different Δ*Z_i_* (*i* = 5~8) and their point-to-point differences with the true value. Since the Δ*Z_i_* (*i* = 5~8) values are extremely small at the order of magnitude of 10^−4^λ, ideal test results can be obtained in each case. It can be seen from Table 3 that the variations of the 5–8 Zernike aberrations of the image plane wavefront are only caused by PG-*θ_z_*, that is, the rotation angle error of the PG during off-axis subaperture scanning. PG-*θ_z_* is relatively easy to control in real experiments, and the probability of it exceeding the maximum value is very small. Considering the coupling effect of PG-*θ_z_* and other alignment and manufacturing errors on the image plane wavefront, even after increasing Δ*Z_i_* (*i* = 5~8) by two orders of magnitude, the PV and RMS of the test errors are still on the order of 1% of the total PV and RMS of the figure error, respectively. In summary, it can be inferred that the PG-*θ_z_* has a small probability of significantly reducing the test results in the real experiment.

### 3.3. Overall Consideration

From the above simulation and analysis, we conclude that:

1. The virtual–real combination SROR method proposed in this paper can eliminate the inherent retrace error caused by the non-null structure of the system and obtain high-precision test results, which demonstrated the feasibility of the algorithm.
2. The measurement accuracy is not affected by the subaperture overlap ratio, and the typical overlap ratio is about 15% to fully cover the SUT.
3. Different alignment and manufacturing errors have different effects on the test results. Supposing that the alignment and manufacturing errors are not considered in the virtual interferometer modeling, it is necessary to precisely align the components, especially LP-Δ*z* and SUT-Δ*z*, which should be reduced to at least 5 µm. Only then can the PV and RMS of the test errors be in the order of 1% of the PV and RMS of the figure error, respectively.

The above control accuracy requirements are difficult to achieve in real experiments, so in order to reduce the control difficulty without reducing the accuracy of the test results, an error correction procedure is suggested to be introduced in the data processing process.

## 4. Preliminary Experiment and Discussions

**Figure 10:**
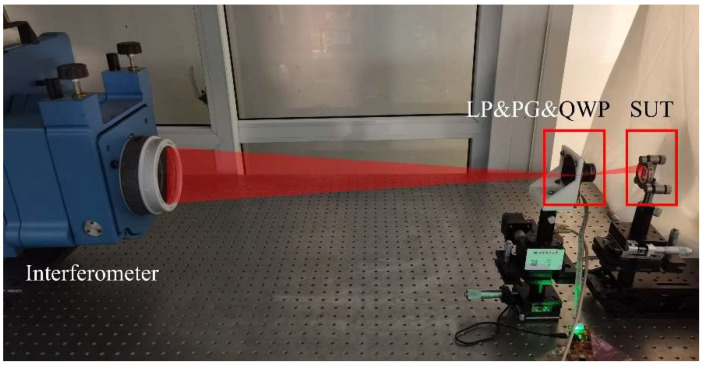
Experimental setup of PG-CSSI. LP is the linear polarizer. PG is the polarization grating. QWP is the λ/4 wave plate. SUT is the surface under test.

**Figure 11:**
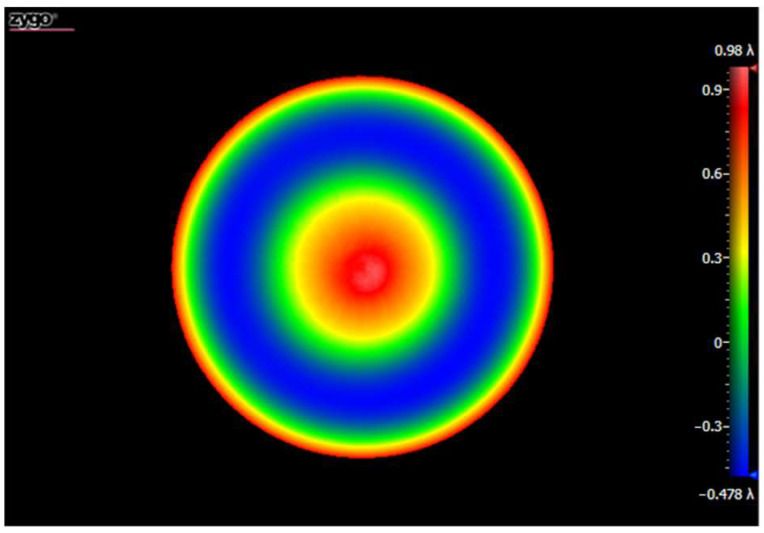
Full-aperture figure error of the spherical SUT.

In order to verify the subaperture scanning performance of the PG-based system, we built an experimental system, as shown in Figure 10. A Zygo interferometer with a 4-inch-aperture F/7.1 TS and a 632.8 nm wavelength was employed for the subaperture test. A LP (FLP20-VIS@LBTEK), PG (customized@LBTEK), and QWP (WPMQ10M-633@Thorlabs) were integrated into a sleeve through the retaining ring and were axially rotated by a rotation mount (ELL14 @Thorlabs). The PG had a space period of 8.035 µm and a sum of ±1st order diffraction efficiency of 98.77%. It had a sandwich structure where a LC layer poured between two windows. The type of thermotropic liquid crystal was nematic liquid crystal. The total thickness of the PG was 2 mm. Due to the PG’s internal insufficient uniformity, in order to minimize the error introduced by such a defect during the scanning process, we placed it near the focus of the TS to minimize the light spot size on the PG, consistent with that shown in Figure 1a. The clear aperture of the spherical SUT was 23.4 mm, and the radius of curvature was 92 mm. The F/# of the SUT was 3.9 and could not be tested with the F/7.1 TS in full aperture. We tested the SUT with a F/3.3 TS and obtained the full-aperture figure error shown in Figure 11. The scanning angle was set to 60°, and the overlap ratio of subapertures was about 32.3%. We did not take special temperature control measures. The ambient temperature during the experiment was in the range of 22 ± 2 °C. The volume of the PG was small, and the single-axis rotation mount was usually much smaller than that of the multi-axis motion-control system, so the volume of the PG-CSSI system was much smaller than that of the traditional SSI system, as shown in Figure 10.

In Section 4.1, we conducted system adjustment experiments, during which we encountered some engineering implementation challenges. In Section 4.2, we performed a subaperture scanning experiment to verify the capability of PG to scan subapertures. In Section 4.3, we propose possible solutions to the engineering challenges.

### 4.1. System Adjustment

**Figure 12:**
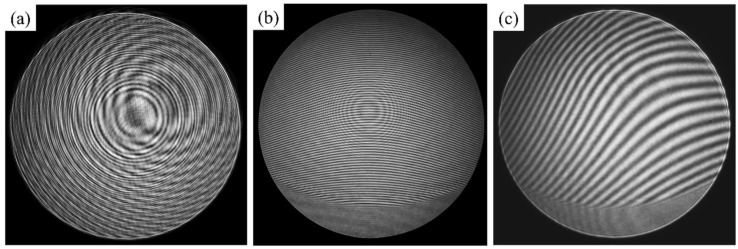
Interferograms captured during the process of system adjustment. The interferograms have been enhanced for better visual effects. (**a**) Stray fringes caused by scanning structure. (**b**) Dense interferogram with aliasing artifacts captured by the detector when the scanning structure was not perpendicular to the optical axis. (**c**) Sparse interferogram after adjusting the SUT.

As mentioned above, the LP, PG, and QWP were integrated into a sleeve through the retaining ring to realize the off-axis subapertures scanning. This structure is hereinafter referred to as the scanning structure. In the experiment, we observed that after the scanning structure was placed perpendicular to the optical axis, a large number of stray fringes appeared in the field of view of the interferometer, and the stray fringes still existed after the SUT was blocked, as shown in Figure 12a. This phenomenon may be because the three components in the scanning structure can be structurally treated as plane-parallel plates. When they were placed perpendicular to the optical axis, the light was reflected multiple times between multiple surfaces and finally back to the interferometer, resulting in stray fringes. In addition, the PG we used has a sandwich structure, that is, the LC layer was poured between the two windows, and the surface of the window in contact with the LC could not be coated with antireflection film, so the reflectivity would be higher than other surfaces.

**Figure 13:**
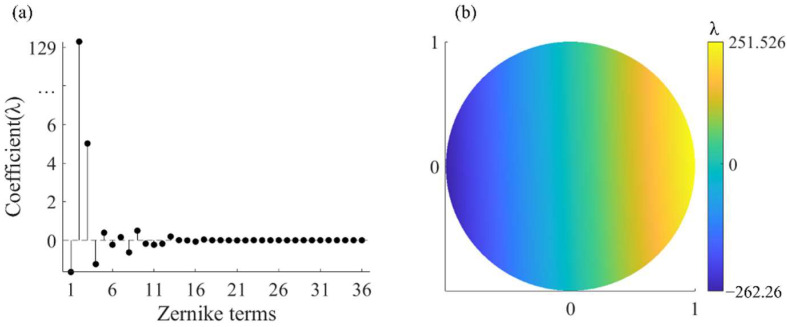
(**a**) Zernike coefficient of image plane wavefront. (**b**) Image plane wavefront.

Therefore, in order to avoid stray fringes caused by multiple reflections, the scanning structure was placed at about 80°50′ to the optical axis. This setup introduced aberrations such as power, tilt, and astigmatism to the transmitted spherical wave, which increased the residual wavefront. When the light reflected by the scanning structure did not return to the interferometer to form stray fringes, the interference fringes generated by the light reflected from the off-axis subaperture on the SUT were too dense, whose frequencies were above the Nyquist frequency of the sensor and aliased to a lower spatial frequency, as shown in Figure 12b. We used 80°50′ as the angle between the first surface of the scanning structure and the optical axis to model PG-CSSI in the ray tracing program and obtained an image plane wavefront with a PV exceeding 500λ, as shown in Figure 13. The corresponding fringe frequency exceeded the Nyquist frequency and could not be correctly sampled by the detector. Obviously, the test result of phase shifting interferometry was incorrect in this case.

In order to achieve sparser fringes that can be correctly demodulated, we adjusted the SUT. Its axial position, *x*-tilt, and *y*-tilt were mainly adjusted. We obtained a sparse interferogram that could be correctly demodulated, as shown in Figure 12c.

### 4.2. Subaperture Scanning Experiment and Analysis

**Figure 14:**
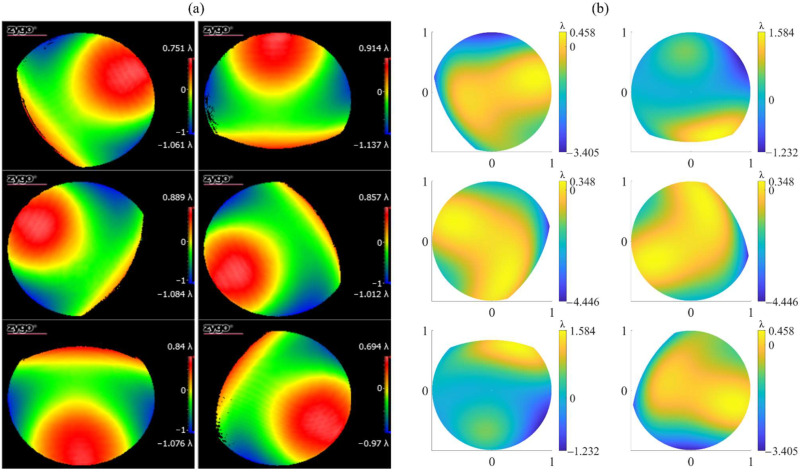
Wavefront maps of the off-axis subapertures in the (**a**) real interferometer and (**b**) virtual interferometer.

As mentioned above, we obtained normally sparse fringes by adjusting the scanning structure and the SUT. We further carried out the subaperture scanning experiment. Using the rotation mount to control the scanning structure to rotate around the axis five times, we obtained the subaperture wavefront maps shown in Figure 14. These results showed that our proposed system could indeed effectively realize the off-axis subaperture scanning only by the axial rotation of the PG.

However, the above operation introduced alignment errors of the scanning structure and the SUT, which meant modeling errors of the virtual interferometer. According to the analysis in Section 3.2, if it caused the image surface wavefront to change, the correct test result might not be obtained by the SROR algorithm. In order to analyze the wavefront changes caused by the alignment error, we added the full-aperture figure error shown in Figure 11 to the virtual interferometer and obtained the predicted wavefront maps of the off-axis subapertures without SUT alignment errors, as shown in Figure 14b. Obviously, the wavefront maps in the real and virtual interferometers were different, which means that adjusting the SUT introduced a large modeling error to the virtual interferometer. Through Zernike fitting, we obtained the differences between the Zernike coefficients of the real and virtual wavefronts, in which the differences of 4 to 8 terms were all in the order of 10^−1^λ, which were much larger than the error tolerance determined in Section 3.2. Therefore, it was difficult to obtain correct test results through the SROR algorithm.

### 4.3. Possible Solution

Based on the above analysis, if the multiple reflections from the surfaces except the SUT did not generate stray interference fringes, the situation that the scanning structure needs to be placed obliquely to the optical axis and a series of subsequent problems caused by it could be directly avoided. A low temporal coherent light source instead of a He-Ne laser in the interferometer can ensure that only the light reflected by the SUT can produce interference fringes with high contrast with the reference light, thereby avoiding stray fringes formed by other reflected light [50]. Since PG was the main source of stray fringes, we can also use PGs based on different structures or materials to avoid stray fringes at the device level.

## 5. Conclusions

This paper proposes a PG-CSSI for small F/# concave surface tests with a large F/# TS. A PG was employed for subaperture scanning instead of the multi-axis motion-control system, which greatly reduced the volume and error source of the CSSI system. The virtual–real combination algorithm eliminated the influence of the retrace error, and the full-aperture surface error distribution was directly obtained without the need for complex stitching algorithms. The feasibility of the PG-CSSI system was verified by simulations, and the tolerance of typical alignment and manufacturing errors of the components were numerically analyzed. When the alignment and manufacturing errors are of the following magnitudes individually, the PV and RMS of the test error are, respectively, 1% of the total PV and RMS of the figure error and below: the lateral translation error of the SUT is less than 0.01 mm, the axial translation error of the LP or the SUT is less than 5 µm, or the axial rotation error of the PG is within ±1°. The subaperture scanning performance of the PG-based system was verified by experiments, but stray interference fringes or unresolvable dense fringes were observed. Based on the above simulations and experiments, combining a low temporal coherence laser, a PG with improved production process and virtual interferometer modeling including alignment and manufacturing error optimization is proposed as the main follow-up research direction.

## Figures and Tables

**Table 1:** The specific parameters of the true figure error and test results.

R/D	K	F/#	Λ **(μm)**	Overlap Ratio	Result		Error	
					PV (λ)	RMS (λ)	PV (×10^−5^ λ)	RMS (×10^−6^ λ)
— ^a^	—	—	—	—	2.4083	0.5829	0	0
1	0	1.8	1.6	14.48%	2.4083	0.5829	1.4846	2.0356
2		3.6	3.125	15.41%			1.4784	2.0358
3		5.3	4.5	14.44%			1.4263	2.0486
4		7.1	6.25	16.91%			1.6270	2.0037
2.5	−2	5.3	4.5	14.44%			1.4801	2.0396
	−1.5						1.4802	2.0406
	−1						1.4807	2.0394
	−0.5						1.4805	2.0400
	0.5						1.4729	2.0300
	1						1.4794	2.0400
	1.5						1.4789	2.0397
	2						1.4857	2.1122

^a^ This line is the true value of the figure error.

**Table 2:** Typical alignment and manufacturing errors of the components.

Error and Its Symbol	Name of the Element			
	LP	PG	QWP	SUT
Axial translation error Δ*z*	0.5 mm	0	0	0.5 mm
Lateral translation error Δ*x*, Δ*y*	0	0	0	0.1 mm
Tilt error *θ_x_*, *θ_y_*	0	0.0225°	0.0225°	0.062°
Axial rotation error *θ_z_*	0	±1°	0	0
Parallelism error *δ_x_*, *δ_y_*	0.00139°	0.00139°	0.00139°	0

**Table 3:** The order of magnitudes (λ) of the variations of the Zernike coefficients describing the off-axis subaperture image plane wavefront caused by each alignment error and manufacturing error.

Element-Error	Δ*Z*_2_ (Tilt)	Δ*Z*_3_ (Tilt)	Δ*Z*_4_ (Power)	Δ*Z*_5_ (Astigmatism)	Δ*Z*_6_ (Astigmatism)	Δ*Z*_7_ (Coma)	Δ*Z*_8_ (Coma)
LP-Δ*z*	— *	—	1	—	—	—	—
LP-*δ_x_*	—	10^−2^	—	—	—	—	—
LP-*δ_y_*	10^−2^	—	—	—	—	—	—
PG-*θ_x_*	10^−1^	10^−1^	—	—	—	—	—
PG-*θ_y_*	10^−1^	10^−1^	—	—	—	—	—
PG-*θ_z_*	10^−4^	10^−4^	—	10^−4^	10^−4^	10^−4^	10^−4^
PG-*δ_x_*	10^−2^	10^−2^	—	—	—	—	—
PG-*δ_y_*	10^−2^	10^−2^	—	—	—	—	—
QWP-*θ_x_*	10^−2^	10^−2^	—	—	—	—	—
QWP-*θ_y_*	10^−2^	10^−2^	—	—	—	—	—
QWP-*δ_x_*	10^−3^	10^−3^	—	—	—	—	—
QWP-*δ_y_*	10^−3^	10^−3^	—	—	—	—	—
SUT-Δ*z*	1	1	1	—	—	—	—
SUT-Δ*x*	10	—	—	—	—	—	—
SUT-Δ*y*	—	10	—	—	—	—	—
SUT-*θ_x_*	—	10	—	—	—	—	—
SUT-*θ_y_*	10	—	—	—	—	—	—

* The item “—” in the table indicates that the effect of the alignment or manufacturing error on the Zernike coefficient is less than 1% compared with other items.

**Table 4:** The figure error test results and errors under different Δ*Z_i_* (*i* = 2~4).

Δ*Z_i_* (λ)		Test Result		Test Error	
		PV (λ)	RMS (λ)	PV (λ)	RMS (λ)
0 (True value)		2.4083	0.5829	0	0
Δ*Z*_2_	10	2.5115	0.5888	0.2360	0.0476
	1	2.4183	0.5840	0.0239	0.0047
Δ*Z*_3_	10	2.6009	0.5077	0.7036	0.1265
	1	2.3953	0.5812	0.0432	0.0070
Δ*Z*_4_	1	3.5673	0.7145	1.2783	0.1820
	10^−2^	2.4190	0.5841	0.0119	0.0018

**Table 5:** The figure error test results and errors under different Δ*Z_i_* (*i* = 5~8).

Δ*Z_i_* (λ)		Test Result		Test Error	
		PV (λ)	RMS (λ)	PV (λ)	RMS (λ)
0 (True value)		2.4083	0.5829	0	0
10^−4^	Δ*Z*_5_	2.4082	0.5829	6.4749 × 10^−4^	7.4275 × 10^−5^
	Δ*Z*_6_	2.4085	0.5829	5.0569 × 10^−4^	6.7166 × 10^−5^
	Δ*Z*_7_	2.4083	0.5829	9.9981 × 10^−4^	1.7070 × 10^−5^
	Δ*Z*_8_	2.4082	0.5829	1.1147 × 10^−4^	1.9517 × 10^−5^
10^−2^	Δ*Z*_5_	2.3980	0.5837	0.0649	0.0074
	Δ*Z*_6_	2.4275	0.5826	0.0495	0.0066
	Δ*Z*_7_	2.4101	0.5831	0.0097	0.0017
	Δ*Z*_8_	2.4051	0.5830	0.0106	0.0019

## Data Availability

Not applicable.
